# Supplementary material for: Human T cells engineered with a leukemia lipid-specific TCR enables donor-unrestricted recognition of CD1c-expressing leukemia
Source: Nat Commun. 2021 Aug 11;12:4844. doi: 10.1038/s41467-021-25223-0 (PMC8358059; doi:10.1038/s41467-021-25223-0)
Supplement: Supplementary file 1 — Supplementary Information [file 41467_2021_25223_MOESM1_ESM.pdf]

SUPPLEMENTARY MATERIALS

SUPPLEMENTARY FIGURES

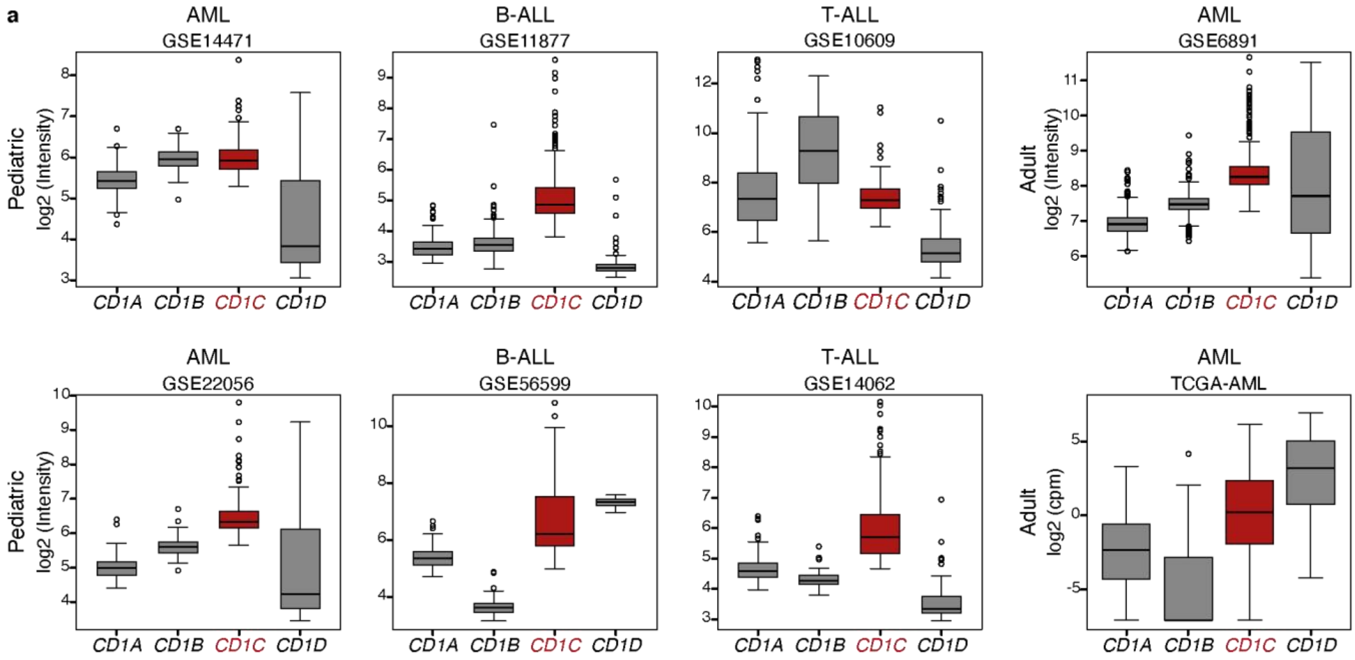

b

Clone Name	TCR V alfa	TCR V beta
DN4.99	TRAV38-2-J44<br>CAYRSPLNTGTASKLTFGTGTRLQVTL	TRBV28-J2.7<br>CASSPWVSSYEQYFGPGTRL
PZP8A6	TRAV26-2-J44<br>CILRDVNTGTASKLTFGTGTRLQVTLD	TRBV4-1-J2.3<br>CASSRLGLSTDTQYFGPGTRLTVL
DN4.2	TRAV26-2-J53<br>CILRLRGGSNYKLTFGKGTLLTVNP	TRBV4-1-J2.1<br>CASSPIMGLAATHNEQFFGPGTRLTVL
P8E3	TRAV38-1-J31<br>CAFANNARLMFGDGTQLVVKP	TRBV28-J1.1<br>CASTDTGNTEAFFGQGTRLTVV
DN7.6.16	TRAV13-1-J28<br>CAAPRGLGVTNSLSGRGPNSRSYQ	TRBV27-J2.1<br>TCASSLVWGTYNEQFFPGTRLTVL

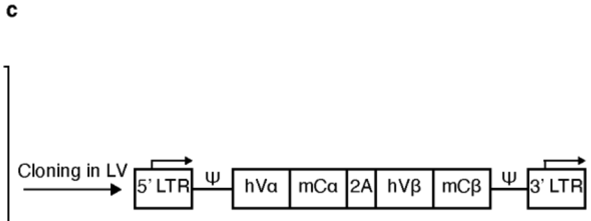

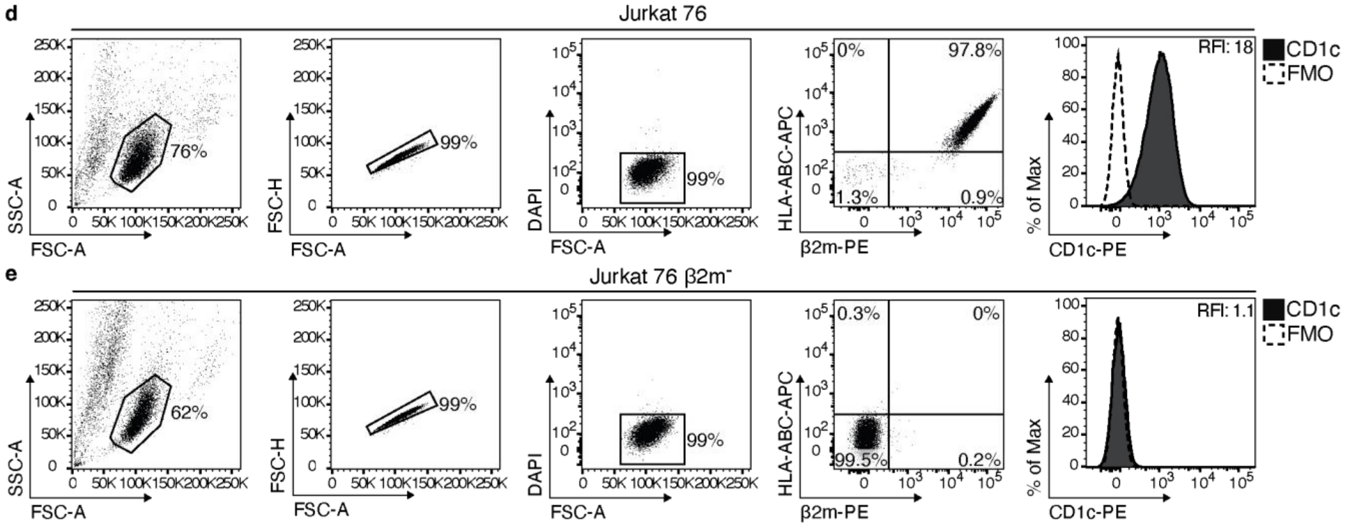

Supplementary Fig. 1: Supporting molecular data for CD1 and TCR expression. a The expression levels of *CD1* genes in datasets not included in Fig.1. In the box plots, the box is enclosed between the first quartile (25th percentile) and the third quartile (75th percentile), the center is represented by the median, the whiskers are defined by 1.5 times the interquartile range (i.e., it is the distance between the upper and lower quartiles). Sample size of each dataset is detailed in Supplementary Table 1. **b** The TCR Vα and Vβ chains expressed by the selected CD1c self-reactive T cell clones. In red are the N regions. DN4.99 and P8E3 T cell clones expressed TRAV38 paired with TRBV28, while DN4.2 and PZP8A6 clones expressed TRAV26 paired with TRBV4. Clone DN7.6.16 expressed Vα and Vβ genes (*TRAV13* and *TRBV27*) different from the other five clones. All clones used different N regions and rearranged different J segments. **c** Representation of the LV vectors carrying cDNA for the CD1c self-reactive TCRs. **d, e** Deletion of CD1c expression from Jurkat 76 cells using CRISPR/Cas9 targeting of the *B2M* gene. Flow cytometry analysis using monoclonal antibodies specific for human HLA-ABC (BioLegend, clone W6/32; diluted 1:100), β2m (BioLegend, clone 2M2; diluted 1:50), and CD1c (Santa Cruz Biotechnology Inc., clone L161, diluted 1:20) on Wild-Type (WT) Jurkat 76 cells (**d**) and after CRISPR/Cas9-mediated editing of the *B2M* gene (**e**), as determined by labeling the cells with the indicated monoclonal antibodies. Dotted histograms represent fluorescence-minus-one (FMO) labeling control. Relative Fluorescence Intensity (RFI) was calculated as the ratio between the intensity of labeling of the sample and control.

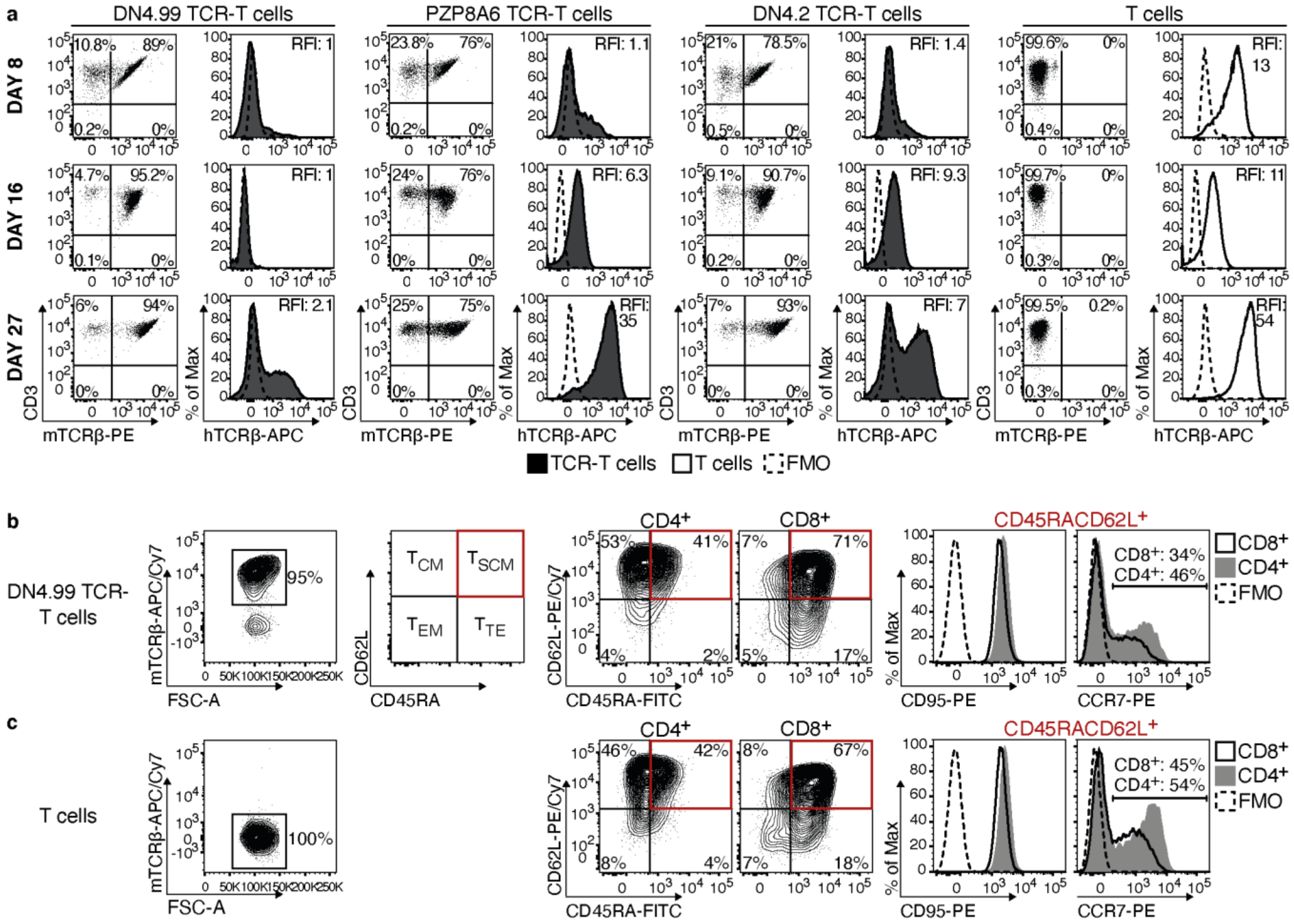

Supplementary Fig. 2: Supporting phenotypical characterization of TCR-transduced T cells. a Transduction of primary polyclonal T cells with the DN4.99 chimeric TCR results in a sustained downregulation of the endogenous TCRs compared to the other methyl-lysophosphatidic acid (mLPA)-specific TCRs. Flow cytometry labeling of the transduced chimeric and endogenous TCRs detected with anti-mouse (m)TCRb and anti-human (h)TCRb monoclonal antibody, respectively, performed at days 8, 16, and 27 from T cell restimulation with the Dynabeads® human T-Activator CD3/CD28 (Thermofisher, Cat# 11131D) at a 3:1 bead:cell ratio. The expression of the endogenous TCRs (white histograms) by the same T cells undergoing the same activation protocol, but without chimeric TCR transduction, is also shown. Black histograms represent TCR-transduced T cells, white histograms represent non-transduced T cells, and dotted histograms represent florescence-minus-one (FMO) labeling control. Relative Fluorescence Intensity (RFI) is shown. **b, c** Distribution of T cell subpopulations ($CCR7^{+}CD95^{+}$ stem cell memory [T_{SCM}], central memory [T_{CM}], effector memory

[T_{EM}] and terminal effector [T_{TE}]) within CD8$^+$ (black histograms) and CD4$^+$ (grey histograms) DN4.99 TCR transduced-T (DN4.99 TCR-T) cells (**b**) and non-transduced T cells (**c**) at day 17 from T cells restimulation with the Dynabeads® human T-Activator CD3/CD28 (Thermofisher, Cat# 11131D) at a 3:1 bead:cell ratio. The flow cytometry analysis was performed labeling the cells with anti-mouse (m)TCRβ-APC/Cy7, anti-human CD8-APC (BioLegend, clone SK1, diluted 1:100), CD62L-PE/Cy7 (BioLegend, clone DREG-56 diluted 1:100), CD45RA-FITC (BioLegend, clone HI100, diluted 1:50); CCR7-PE (BioLegend, clone G043H7, diluted 1:50;), CD95-PE (BioLegend, clone DX2, diluted 1:100) and CD4-V500 (BD, clone RPTA-4, diluted 1:100;) monoclonal antibodies. Dotted histograms represent florescence-minus-one (FMO) labeling control.

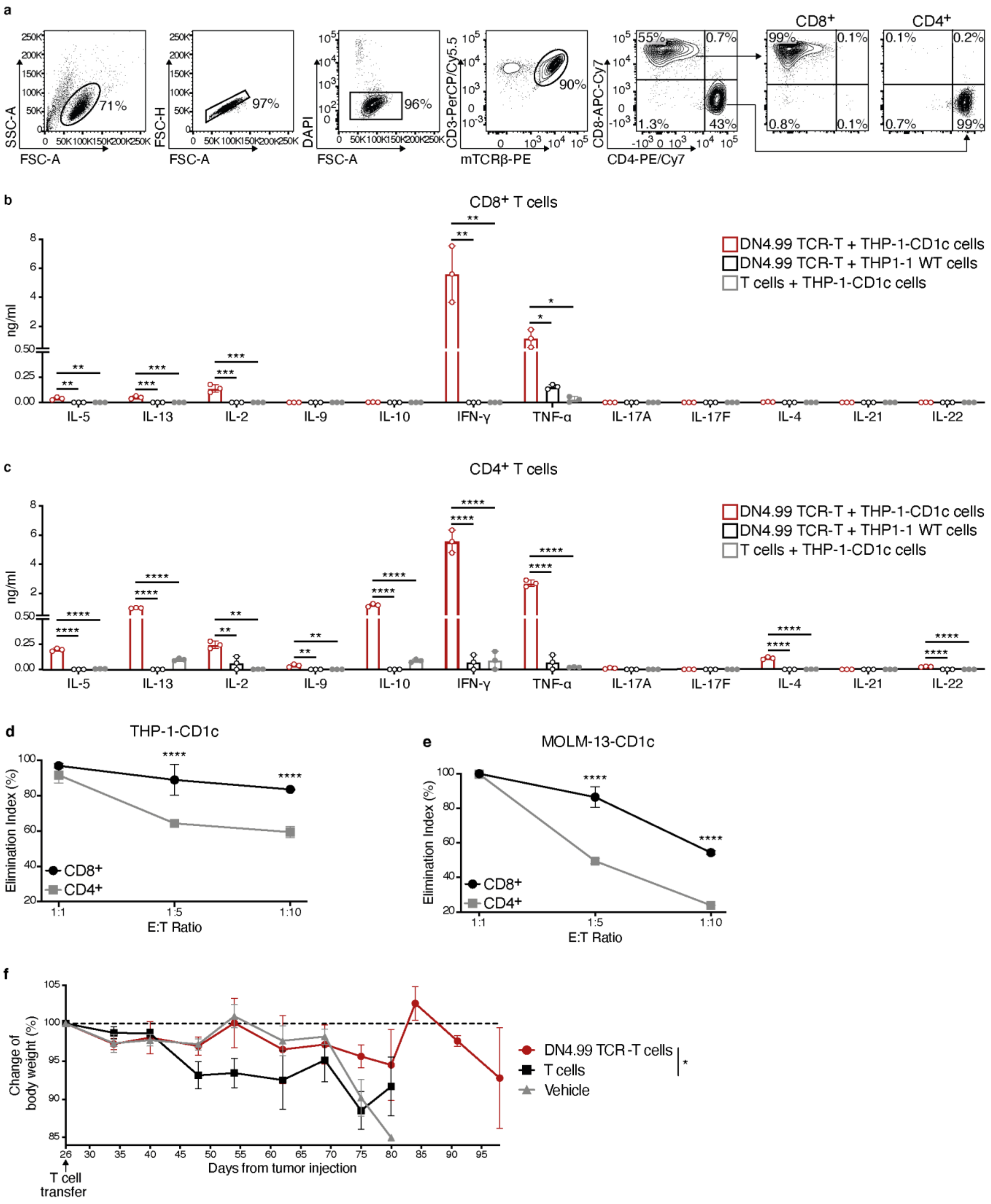

Supplementary Fig. 3: Supporting functional characterization of TCR-transduced T cells in vitro and in vivo. a-c CD8^{+} and CD4^{+} DN4.99 TCR-transduced T (DN4.99 TCR-T) cells are polyfunctional. **a** Primary T cells were transduced with the lead methyl-lysophosphatidic acid

(mLPA)-specific DN4.99 TCR and separated by immunomagnetic cell sorting using CD4 Isolation Kit (Miltenyi Biotec, Cat# 130-096-533) into CD8$^+$ and CD4$^+$ subsets at ≥99% final purity. CD8$^+$ (**b)** and CD4$^+$ (**c)** DN4.99 TCR-T cells were individually assessed in vitro against THP-1-CD1c cells (red bars) or THP-1-Wild-Type (WT) cells (grey bars) at Effector:Target (E:T) ratio 1:1 to analyze, after 48 hours of co-culture, the cytokine release with Th Cytokine Panel LEGENDPlex (Biolegend, Cat# 740722) according to manufacturer's instructions. CD8$^+$ or CD4$^+$ non-transduced T cells assessed against THP-1-CD1c cells (grey bars) were used as further control. Data are represented as mean ± SD. Shown is one of two independent experiments with n=3 replicates per experiment. *P < 0.05; **P < 0.01; ***P < 0.001; ****P < 0.0001 determined by Ordinary one-way ANOVA followed by Tukey's multiple comparison test. **d, e** CD8$^+$ and CD4$^+$ DN4.99 TCR-T cells differentially kill primary CD1c$^+$ leukemia cell lines in vitro. CD8$^+$ (black dots) and CD4$^+$ (grey squares) subsets (obtained as described for panels a and b), were individually assessed in vitro for the ability to kill CD1c-expressing THP-1 (**d**) and MOLM-13 (**e**) acute leukemia cell lines at the indicated E:T ratio. The killing of the target cells was determined by flow cytometry labeling and expressed as Elimination Index, as described in Methods. Data are represented as mean ± SD. Shown is one of two consistent independently performed experiments with n=3 replicates per experiment. ****P < 0.0001 determined by Ordinary one-way ANOVA followed by Tukey's multiple comparison test. **f** DN4.99 TCR-T cell transfer doesn't induce Graft-versus-Host-Disease in mice injected with THP-1-CD1c cells. Mice receiving: DN4.99 TCR-T cells, red dots; T cells, black squares; and vehicle, grey triangles. Body weight was monitored at several time points after T cell injection. Data are represented as mean ± SD. Shown is one of two independent experiments with n=3 replicates per experiment. *P = 0.0396 determined on AUC by two-tailed unpaired t-test.

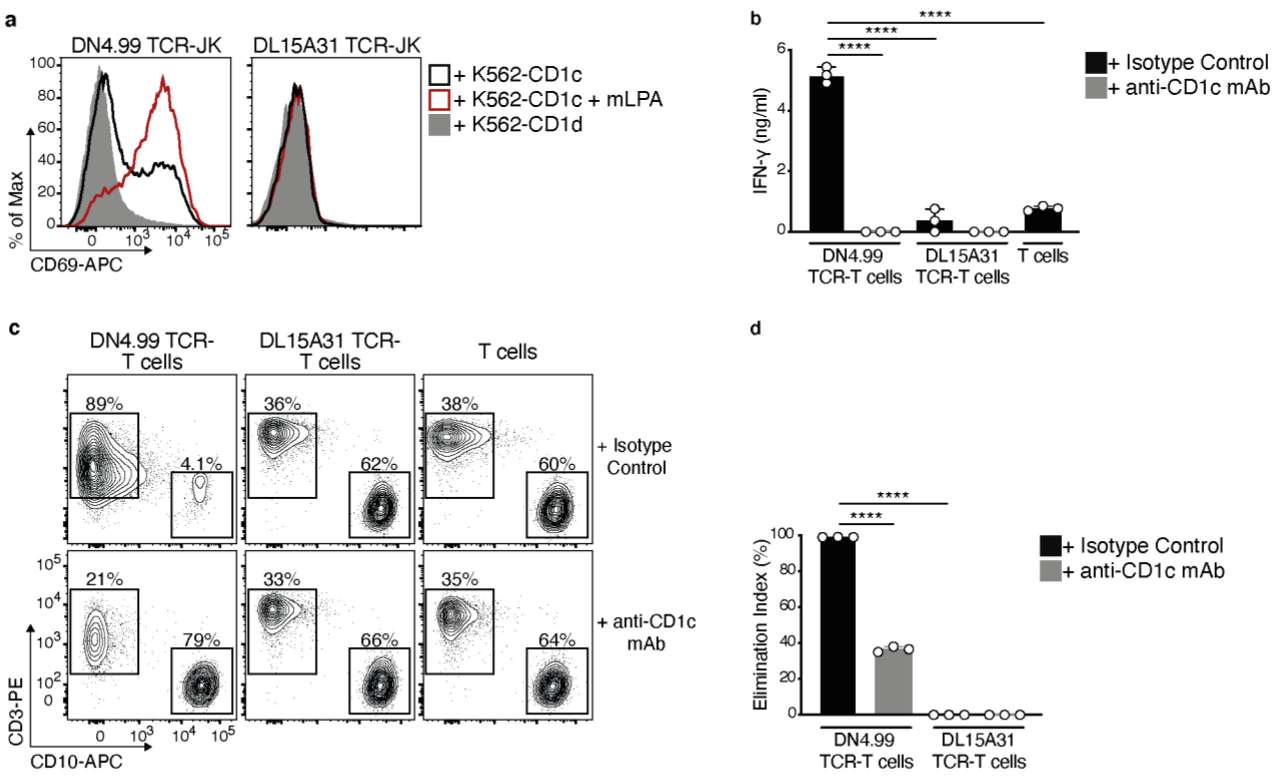

Supplementary Fig. 4: DL15A31 TCR is not CD1c-self-reactive and not mLPA (methyl-lysophosphatidic acid) specific. a Jurkat 76 β_2m^- cells transduced with DN4.99 TCR (DN4.99 TCR-JK) or DL15A31 TCR (DL15A31 TCR-JK) were cultured at a 1:1 Effector:Target (E:T) ratio with $5x10^4$ K562-CD1c cells loaded (red histograms) or not (black histograms) with 1.5 ng/ml of methyl-lysophosphatidic acid (mLPA). K562-CD1d cells (grey histograms) were used as a negative control. Jurkat cell activation after overnight co-culture was assessed as increased intensity of CD69 expression as measured by flow cytometry (Mean Fluorescence Intensity, MFI, reported)**. b-d** DN4.99 TCR-T cells, but not DL15A31-TCR T cells, efficiently recognize and kill $CD1c^+$ NALM-6 cells in vitro. **b** Recognition of NALM-6-CD1c cells by primary T cells transduced with the DN4.99, or DL15A31 TCRs or not-transduced upon co-culture at a 1:1 E:T ratio in the presence or in the absence of anti-CD1c monoclonal antibody (20µg/mL). After 48h supernatants were collected and IFN-γ production was measured by ELISA. Data are represented as mean ± SD. Shown is one of two independent experiments with n=3 replicates per experiment. ****P < 0.0001 determined by Ordinary one-way ANOVA followed by Tukey's multiple comparison test. **c, d** Representative killing of NALM-6-CD1c cells by DN4.99 TCR-T cells. After 72h of co-culture at a 1:1 E:T ratio,

killing of the NALM-6-CD1c cell line was determined by flow cytometry labeling (**c**) and expressed as elimination index (**d**), as described in Methods. Histogram data are represented as mean ± SD. Results are representative of two independent experiments with n=3 replicates per experiment. ****P < 0.0001 determined by Ordinary one-way ANOVA followed by Tukey's multiple comparison test.

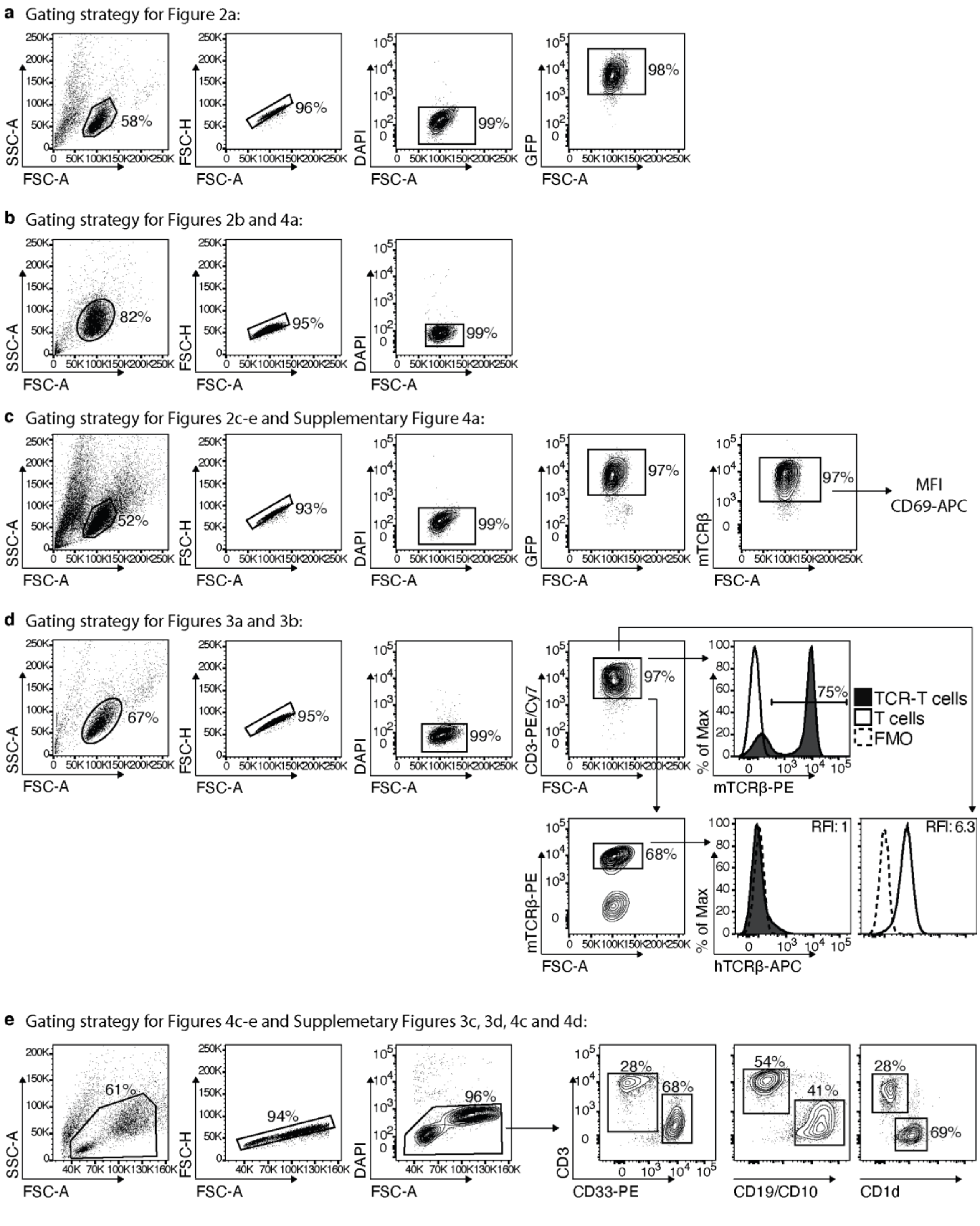

Supplementary Fig. 5: Gating strategy for Figures 2-4 and Supplementary Figures 3 and 4. a Gating strategy for Fig. 2a to determine the expression of the transduced chimeric TCRs on Jurkat 76 cells. **b** Gating strategy for Fig. 2b and Fig. 4a to determine the CD1c expression on the leukemia cell lines. **c** Gating strategy for Fig. 2c-e and Supplementary Fig. 4a to determine the mean fluorescence intensity (MFI) of CD69 on TCR-transduced Jurkat 76 cells. **d** Gating strategy for Fig. 3a and 3b to

determine the expression of transduced chimeric TCRs (Fig. 3a) and the endogenous TCR (Fig 3b) on transduced primary T cells. Chimeric TCR expression on transduced (black histograms) and non-transduced (white histograms) primary polyclonal T cells was determined by labeling the $CD3^+$ cells with anti-mouse TCRβ (mTCRβ) monoclonal antibody. The endogenous human TCR expression in chimeric TCR-transduced T cells was determined by labeling the $mTCR\beta^+$ transduced T cells with anti-human TCRβ (hTCRβ) monoclonal antibody. The endogenous human TCR expression in non-transduced T cells (white histograms) was evaluated in the $CD3^+$ cells. **e** Gating strategy for Fig. 4c-e and Supplementary Figures 3c-d and 4c-d to assess the killing of CD1c-expressing leukemia cell lines. Effector T cells were labelled with anti-human CD3 monoclonal antibody; Target cells were identified as follow: AML (THP-1, MOLM-13, K562) as $CD33^+$; B-ALL (NALM-6, CCRF-SB) and B lymphoblatoid cells (C1R) as $CD19^+$ or $CD10^+$; T-ALL (MOLT-4) as $CD1d^+$.

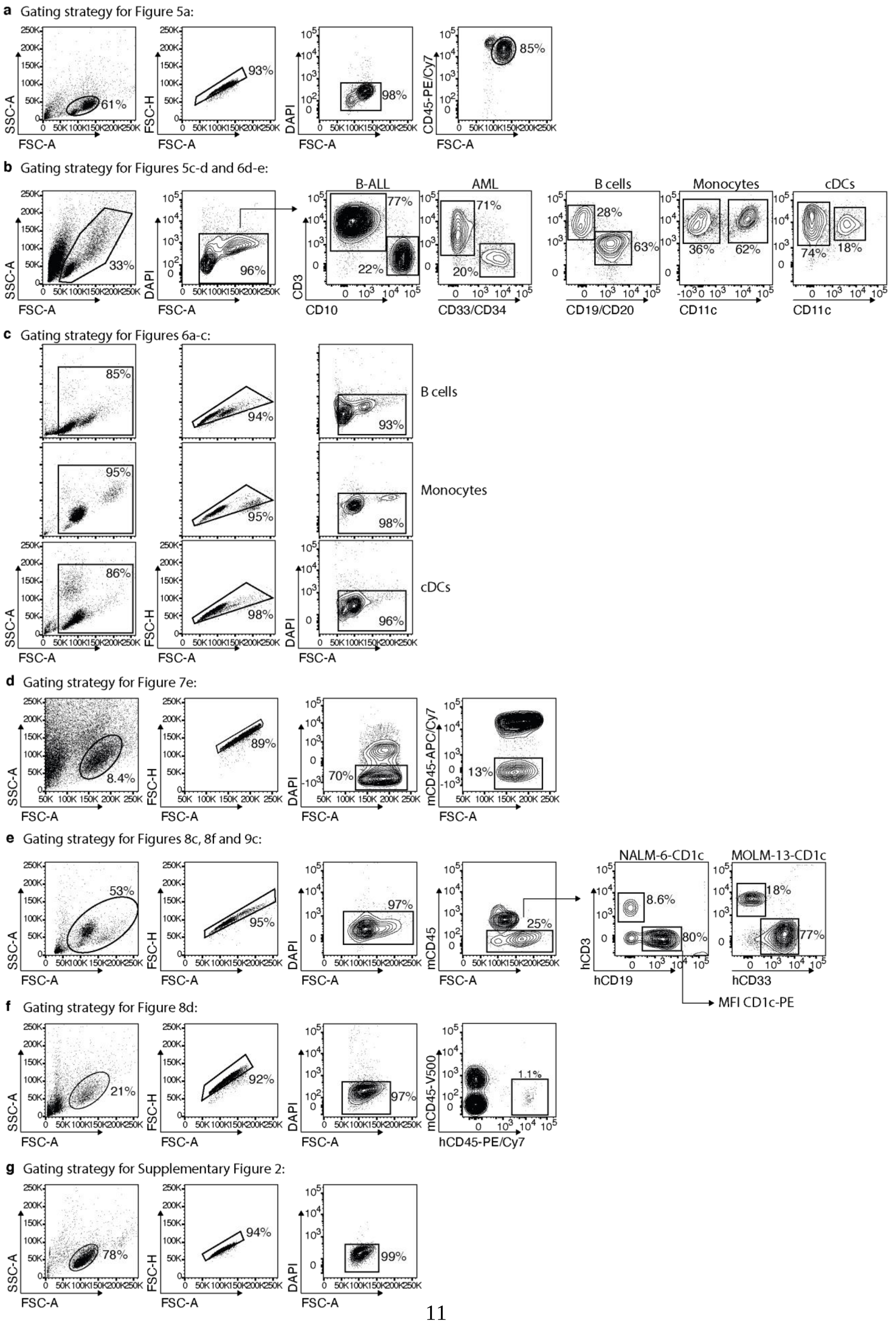
a Gating strategy for Figure 5a:
SSC-A
FSC-A
61%
FSC-H
93%
DAPI
98%
CD45-PE/Cy7
85%
b Gating strategy for Figures 5c-d and 6d-e:
33%
96%
B-ALL
CD3
CD10
77%
22%
AML
CD33/CD34
71%
20%
B cells
CD19/CD20
28%
63%
Monocytes
CD11c
36%
62%
cDCs
74%
18%
c Gating strategy for Figures 6a-c:
85%
94%
93%
B cells
95%
95%
98%
Monocytes
86%
98%
96%
cDCs
d Gating strategy for Figure 7e:
8.4%
89%
70%
mCD45-APC/Cy7
13%
e Gating strategy for Figures 8c, 8f and 9c:
53%
95%
97%
mCD45
25%
NALM-6-CD1c
hCD3
hCD19
8.6%
80%
MOLM-13-CD1c
hCD33
18%
77%
MFI CD1c-PE
f Gating strategy for Figure 8d:
21%
92%
97%
mCD45-V500
hCD45-PE/Cy7
1.1%
g Gating strategy for Supplementary Figure 2:
78%
94%
99%

Supplementary Fig. 6: Gating strategy for Figures 5-8 and Supplementary Figure 2. a Gating strategy for Fig. 5a to determine the expression of CD1c on primary leukemia blasts. **b** Gating strategy for Fig. 5c-d and Fig. 6d-e to assess the killing of both primary CD1c^{+} acute leukemia blasts (Fig. 5c-d), and normal CD1c^{+} blood cells (Fig. 6d-e). Effector T cells were labelled with anti-human CD3 monoclonal antibody; Target cells were identified as follow: B-ALL as CD10^{+}; AML as CD33^{+} or CD34^{+} depending on the AML blasts phenotype; B cells as CD19^{+} or CD20^{+}; monocytes and circulating dendritic cells (cDCs) as CD11c^{+}. **c** Gating strategy for Fig. 5a-e to assess the normal B cells, monocytes and cDCs purification and the CD1c expression. **d** Gating strategy for figure 7e to determine at sacrifice the persistence of DN4.99 TCR-transduced T cells in the liver of NOD.Cg-Prkdcscid IL- 2rgtm1Wjl/SzJ (NSG) mice that received intravenous injection of THP-1-CD1c leukemic cells. **e** Gating strategy for Fig. 8c, 8f and 9c to determine both the percentage of tumors cells and their CD1c expression (Fig. 8), and the percentage of transferred DN4.99 TCR-transduced T cells (Fig. 9) in the peripheral blood of NOD.Cg-Prkdcscid IL-2rgtm1Wjl/SzJ (NSG) tumor bearing mice. Human T cells were identified as mouse (m)CD45^{-} human (h)CD3^{+}; human leukemia cells were identified either as human (h)CD19^{+} (the B-ALL NALM-6-CD1c) or as human (h)CD33^{+} (the AML MOLM-13-CD1c). The mean fluorescence intensity (MFI) of CD1c were evaluated on CD19^{+} NALM-6-CD1c leukemic cells. **f** Gating strategy for Fig. 8d to assess the presence of DN4.99 TCR-T cells in the bone marrow of NOD.Cg-Prkdcscid IL- 2rgtm1Wjl/SzJ (NSG) NALM-6 CD1c bearing mice. The human cells were identified as mouse (m)CD45^{-} human (h)CD45^{+}. **g** Gating strategy for Supplementary Fig. 2 for the phenotypical characterization of TCR-transduced T cells.

SUPPLEMENTARY TABLES

Supplementary Table 1: Leukemia list of the datasets investigated for CD1 expression. Leukemia type, number of cases, and Gene Expression Omnibus (GEO) or The Cancer Genome Atlas program (TCGA) ID of the datasets investigated for CD1 expression. AML: Acute Myeloid Leukemia; B-ALL: B-cell Acute Lymphoblastic Leukemia; T-ALL: T-cell Acute Lymphoblastic Leukemia.

Leukemia type		#cases	ID	Reference
AML	adult	151	TCGA-LAML https://portal.gdc.cancer.gov/projects/TCGA-LAML	ref[1]
		242	GSE12417 https://www.ncbi.nlm.nih.gov/geo/query/acc.cgi?acc=GSE12417	ref[2]
		460	GSE6891 https://www.ncbi.nlm.nih.gov/geo/query/acc.cgi?acc=GSE6891	ref[3]
	pediatric	237	GSE17855 https://www.ncbi.nlm.nih.gov/geo/query/acc.cgi?acc=GSE17855	ref[4]
		89	GSE14471 https://www.ncbi.nlm.nih.gov/geo/query/acc.cgi?acc=GSE14471	ref[5]
		98	GSE22056 https://www.ncbi.nlm.nih.gov/geo/query/acc.cgi?acc=GSE22056	ref[3]
B-ALL	adult	27	GSE18497 https://www.ncbi.nlm.nih.gov/geo/query/acc.cgi?acc=GSE18497	ref[6]
		19	GSE14834 https://www.ncbi.nlm.nih.gov/geo/query/acc.cgi?acc=GSM371767	ref[7]
	pediatric	197	GSE13576 https://www.ncbi.nlm.nih.gov/geo/query/acc.cgi?acc=GSE13576	ref[8]
		207	GSE11877 https://www.ncbi.nlm.nih.gov/geo/query/acc.cgi?acc=GSE11877	ref[9]
		81	GSE56599 https://www.ncbi.nlm.nih.gov/geo/query/acc.cgi?acc=GSE56599	ref[10]
T-ALL	adult	92	GSE14618 https://www.ncbi.nlm.nih.gov/geo/query/acc.cgi?acc=GSE14618	ref[11]
		14	GSE18497 https://www.ncbi.nlm.nih.gov/geo/query/acc.cgi?acc=GSE18497	ref[6]

	pediatric	43	GSE50999 https://www.ncbi.nlm.nih.gov/geo/query/acc.cgi?acc=GSE50999	ref[12]
		91	GSE10609 https://www.ncbi.nlm.nih.gov/geo/query/acc.cgi?acc=GSE10609	ref[13]
		90	GSE14062 https://www.ncbi.nlm.nih.gov/geo/query/acc.cgi?acc=GSE14062	ref[14]

Supplementary Table 2: Differential expression analysis of CD1c in 31 tumors from the TCGA project and their corresponding normal tissue. The normal cohort is a sum of normal samples from both TCGA and GTEx databases. Modulation was specified for significant datasets only. Up: the gene is significantly more highly expressed in the tumor tissue compared to normal tissue; down: the gene is significantly less expressed in tumor tissue compared to normal tissue; ns: p-value > 0.01 according to one-way ANOVA.

TCGA project				GTEx project		Statistics	
ID	Tumor	#tumor samples	#normal samples	Tissue	#normal samples	modulation	p-value
ACC	Adrenocortical carcinoma	77	-	Adrenal Gland	128	*ns*	*ns*
BLCA	Bladder Urothelial Carcinoma	404	19	Bladder	9	*ns*	*ns*
BRCA	Breast invasive carcinoma	1085	112	Breast	179	*ns*	*ns*
CESC	Cervical squamous cell carcinoma and endocervical adenocarcinoma	306	3	Cervix Uteri	10	*ns*	*ns*
CHOL	Cholangio carcinoma	36	9	-	-	*ns*	*ns*
COAD	Colon adenocarcinoma	275	41	Colon	308	*ns*	*ns*
DLBC	Lymphoid Neoplasm Diffuse	47	-	Blood	337	up	<0.01

	Large B-cell Lymphoma[15]						
ESCA	Esophageal carcinoma	182	13	Esophagus	273	*ns*	*ns*
GBM	Glioblastoma multiforme	163	-	Brain	207	*ns*	*ns*
HNSC	Head and Neck squamous cell carcinoma	519	44	-	-	*ns*	*ns*
KICH	Kidney Chromophobe	66	25	Kidney	28	*ns*	*ns*
KIRC	Kidney renal clear cell carcinoma	523	72	Kidney	28	*ns*	*ns*
KIRP	Kidney renal papillary cell carcinoma	286	32	Kidney	28	*ns*	*ns*
LAML	Acute Myeloid Leukemia[1]	173	-	Bone Marrow	70	up	<0.01
LGG	Brain Lower Grade Glioma	518	-	Brain	207	*ns*	*ns*
LIHC	Liver hepatocellular carcinoma	369	50	Liver	110	*ns*	*ns*
LUAD	Lung adenocarcinoma	483	59	Lung	288	*ns*	*ns*

LUSC	Lung squamous cell carcinoma	486	50	Lung	288	*ns*	*ns*
OV	Ovarian serous cystadenocarcinoma	426	-	Ovary	88	*ns*	*ns*
PAAD	Pancreatic adenocarcinoma	179	4	Pancreas	167	up	<0.01
PCPG	Pheochromocytoma and Paraganglioma	182	3	-	-	*ns*	*ns*
PRAD	Prostate adenocarcinoma	492	52	Prostate	100	*ns*	*ns*
READ	Rectum adenocarcinoma	92	10	Colon	308	*ns*	*ns*
SARC	Sarcoma	262	2	-	-	*ns*	*ns*
SKCM	Skin Cutaneous Melanoma	461	1	Skin	557	down	<0.01
STAD	Stomach adenocarcinoma	408	36	Stomach	175	*ns*	*ns*
TGCT	Testicular Germ Cell Tumors	137	-	Testis	165	*ns*	*ns*
THCA	Thyroid carcinoma	512	59	Thyroid	278	*ns*	*ns*
THYM	Thymoma	118	2	Blood	337	up	<0.01

UCEC	Uterine Corpus Endometrial Carcinoma	174	13	Uterus	78	*ns*	*ns*
UCS	Uterine Carcinosarcoma	57	-	Uterus	78	*ns*	*ns*

Supplementary Table 3: Primers used to clone the CD1c cDNA. Underlined the Kozak sequence, in red ATG and stop codons.

Name	Sequence
Up-hCD1c	5’CCGCCATGCTGTTTCTGCAGTTTCTGC3’
Dw-hCD1c	5’CCTACAGGATGTCCTGATATGAGCAG3’

SUPPLEMENTARY REFERENCES

1. Cancer Genome Atlas Research Network *et al.* Genomic and epigenomic landscapes of adult de novo acute myeloid leukemia. *N Engl J Med* **368,** 2059–2074 (2013).
2. Metzeler, K. H. *et al.* An 86-probe-set gene-expression signature predicts survival in cytogenetically normal acute myeloid leukemia. *Blood* **112,** 4193–4201 (2008).
3. Verhaak, R. G. W. *et al.* Prediction of molecular subtypes in acute myeloid leukemia based on gene expression profiling. *Haematologica* **94,** 131–134 (2009).
4. Balgobind, B. V. *et al.* Evaluation of gene expression signatures predictive of cytogenetic and molecular subtypes of pediatric acute myeloid leukemia. *Haematologica* **96,** 221–230 (2011).
5. Radtke, I. *et al.* Genomic analysis reveals few genetic alterations in pediatric acute myeloid leukemia. *Proc Natl Acad Sci USA* **106,** 12944–12949 (2009).
6. Staal, F. J. T. *et al.* Genome-wide expression analysis of paired diagnosis-relapse samples in ALL indicates involvement of pathways related to DNA replication, cell cycle and DNA repair, independent of immune phenotype. *Leukemia* **24,** 491–499 (2010).
7. Fulci, V. *et al.* Characterization of B- and T-lineage acute lymphoblastic leukemia by integrated analysis of MicroRNA and mRNA expression profiles. *Genes Chromosomes Cancer* **48,** 1069–1082 (2009).
8. Meyer, L. H. *et al.* Early relapse in ALL is identified by time to leukemia in NOD/SCID mice and is characterized by a gene signature involving survival pathways. *Cancer Cell* **19,** 206–217 (2011).
9. Kang, H. *et al.* Gene expression classifiers for relapse-free survival and minimal residual disease improve risk classification and outcome prediction in pediatric B-precursor acute lymphoblastic leukemia. *Blood* **115,** 1394–1405 (2010).

10. Lee, S.-T. *et al.* Epigenetic remodeling in B-cell acute lymphoblastic leukemia occurs in two tracks and employs embryonic stem cell-like signatures. *Nucleic Acids Res* **43,** 2590–2602 (2015).

11. Winter, S. S. *et al.* Identification of genomic classifiers that distinguish induction failure in T-lineage acute lymphoblastic leukemia: a report from the Children's Oncology Group. *Blood* **110,** 1429–1438 (2007).

12. Silveira, A. B. *et al.* PI3K inhibition synergizes with glucocorticoids but antagonizes with methotrexate in T-cell acute lymphoblastic leukemia. *Oncotarget* **6,** 13105–13118 (2015).

13. Van Vlierberghe, P. *et al.* The recurrent SET-NUP214 fusion as a new HOXA activation mechanism in pediatric T-cell acute lymphoblastic leukemia. *Blood* **111,** 4668–4680 (2008).

14. Zangrando, A., Dell'orto, M. C., Kronnie, Te, G. & Basso, G. MLL rearrangements in pediatric acute lymphoblastic and myeloblastic leukemias: MLL specific and lineage specific signatures. *BMC Med Genomics* **2,** 36–12 (2009).

15. Schmitz, R. *et al.* Genetics and pathogenesis of Diffuse Large B-Cell Lymphoma. *N Engl J Med* **378,** 1396–1407 (2018).
