# Supplementary material for: Human T cells engineered with a leukemia lipid-specific TCR enables donor-unrestricted recognition of CD1c-expressing leukemia
Source: Nat Commun. 2021 Aug 11;12:4844. doi: 10.1038/s41467-021-25223-0 (PMC8358059; doi:10.1038/s41467-021-25223-0)
Supplement: Supplementary file 3 — Reporting Summary [file 41467_2021_25223_MOESM3_ESM.pdf]

nature research

Corresponding author(s): Giulia Casorati
Paolo Dellabona

Last updated by author(s): Jul 6, 2021

Reporting Summary

Nature Research wishes to improve the reproducibility of the work that we publish. This form provides structure for consistency and transparency in reporting. For further information on Nature Research policies, see our Editorial Policies and the Editorial Policy Checklist.

Statistics

For all statistical analyses, confirm that the following items are present in the figure legend, table legend, main text, or Methods section.

n/a	Confirmed	
☐	☒	The exact sample size (*n*) for each experimental group/condition, given as a discrete number and unit of measurement
☐	☒	A statement on whether measurements were taken from distinct samples or whether the same sample was measured repeatedly
☐	☒	The statistical test(s) used AND whether they are one- or two-sided<br>*Only common tests should be described solely by name; describe more complex techniques in the Methods section.*
☒	☐	A description of all covariates tested
☐	☒	A description of any assumptions or corrections, such as tests of normality and adjustment for multiple comparisons
☐	☒	A full description of the statistical parameters including central tendency (e.g. means) or other basic estimates (e.g. regression coefficient) AND variation (e.g. standard deviation) or associated estimates of uncertainty (e.g. confidence intervals)
☐	☒	For null hypothesis testing, the test statistic (e.g. *F*, *t*, *r*) with confidence intervals, effect sizes, degrees of freedom and *P* value noted<br>*Give P values as exact values whenever suitable.*
☒	☐	For Bayesian analysis, information on the choice of priors and Markov chain Monte Carlo settings
☒	☐	For hierarchical and complex designs, identification of the appropriate level for tests and full reporting of outcomes
☒	☐	Estimates of effect sizes (e.g. Cohen's *d*, Pearson's *r*), indicating how they were calculated

Our web collection on statistics for biologists contains articles on many of the points above.

Software and code

Policy information about availability of computer code

Data collection	Microarray raw data were downloaded as CEL files from GEO using GEOquery (v2.46) package; Raw data for the TCGA-LAML datasaet were downloaded as row counts from TCGA repository using TCGAbiolinks package (v2.15);<br>BD FACSDiva (v8.0.2) for flow cytometer (FACS Canto II, BD); BIO-RAD Microplate Manager software (v6.3) for ELISA reader (iMark Microplate Reader, BIO-RAD); MikroWin 2000 (v4.41) for luminometer (BertholdTech Mithras).
Data analysis	The analyses of gene expression data were performed in R (v3.4.4) using Bioconductor libaries of BioC 3.6 and R statistical packages: the ComBat function of the SVA (v. 3.26.0) package for batch correction; Affy (v1.56) package to normalized raw intensity signals of CEL files; edgeR R package (v. 3.20.0) to normalize TCGA data; GEPIA tool (v1.0) for the differential analysis of CD1c expression between TCGA tumor and its corresponding normal tissue from GTEx.<br>FlowJo (BD, v10.6) for analysis of flow cytometry data; GraphPad Prism (v8) for statistics.

For manuscripts utilizing custom algorithms or software that are central to the research but not yet described in published literature, software must be made available to editors and reviewers. We strongly encourage code deposition in a community repository (e.g. GitHub). See the Nature Research guidelines for submitting code & software for further information.

Data

Policy information about availability of data

All manuscripts must include a data availability statement. This statement should provide the following information, where applicable:

- Accession codes, unique identifiers, or web links for publicly available datasets
- A list of figures that have associated raw data
- A description of any restrictions on data availability

The authors declare that all data supporting the findings of this study are available within the paper and its supplementary information files or from the

corresponding authors upon reasonable request. Microarray raw gene expression data (.CEL files) of all series listed in Supplementary Table 1 are available in Gene Expression Omnibus (https://www.ncbi.nlm.nih.gov/geo/). Raw data of the TCGA datasets are freely accessible at TCGA (https://www.cancer.gov/about-nci/organization/ccg/research/structural-genomics/tcga) and they have been download from the repository using the TCGAbiolinks R package. The dataset IDs are detailed in Supplementary Table 1. Source data for all figures are provided with the paper, except for Figure 1c since the publicly-available online tool utilized to generate the graphs (http://gepia.cancer-pku.cn, as indicated in methods) does not make them available.

Field-specific reporting

Please select the one below that is the best fit for your research. If you are not sure, read the appropriate sections before making your selection.

- [x] Life sciences
- [ ] Behavioural & social sciences
- [ ] Ecological, evolutionary & environmental sciences

For a reference copy of the document with all sections, see nature.com/documents/nr-reporting-summary-flat.pdf

Life sciences study design

All studies must disclose on these points even when the disclosure is negative.

Sample size	Although statistical methods were not used to predetermine sample sizes, they were chosen on the basis of estimates from pilot experiments and were similar to those generally employed and accepted in the field. All sample sizes are shown in the figure legends of our manuscript. In the in vivo experiments, numbers of animals are similar to those generally used in similar studies and were sufficient to support our conclusions with statistical significance.
Data exclusions	No data were excluded from anlysis.
Replication	We have performed all experiments shown in our manuscripts at least twice. Each experiment in vitro contained 3 independent replicates per sample. Each experiment in vivo was performed with n=5-10 mice/group. Experimental repetitions were each done utilizing independent T cell lines giving comparable results. Exact numbers of biologically independent experimental repetition are stated in the manuscript.
Randomization	Blood samples from which primary leukocytes (T cells, B cells, monocytes and DCs) were purified were obtained from randomized healthy donors from the Ospedale San Raffaele.<br>For the in vitro recognition experiments, samples were allocated into the experimental groups according to their cell type: established leukemia cell lines; primary circulating leukemia blasts; primary circulating normal leukocytes (B cell, monocytes, cDCs).<br>Leukemia bearing mice were always randomized in groups before receiving the indicated treatments. Mice of the same sex and age were used to control of covariates.
Blinding	In vivo experiments were performed unblinded due to requirements and regulations of our animal facility, as the investigator has to personally treat the mice of concern. For the experiments in vitro, the investigators were not blinded because treatments and the data collection were performed by the same people. However, the experimental repetitions were performed at least by two scientists.

Reporting for specific materials, systems and methods

We require information from authors about some types of materials, experimental systems and methods used in many studies. Here, indicate whether each material, system or method listed is relevant to your study. If you are not sure if a list item applies to your research, read the appropriate section before selecting a response.

Materials & experimental systems

n/a	Involved in the study	
[ ]	[x]	Antibodies
[ ]	[x]	Eukaryotic cell lines
[x]	[ ]	Palaeontology and archaeology
[ ]	[x]	Animals and other organisms
[ ]	[x]	Human research participants
[x]	[ ]	Clinical data
[x]	[ ]	Dual use research of concern

Methods

n/a	Involved in the study	
[x]	[ ]	ChIP-seq
[ ]	[x]	Flow cytometry
[x]	[ ]	MRI-based neuroimaging

Antibodies

Antibodies used	CD1c expression was detected with the anti-CD1c PE mAb from Santa Cruz Biotechnology Inc., Cat# sc-18886, clone L161, or the anti-CD1c PE from BioLegend, Cat# 331506, clone L161 only for B-ALL-31. The isotype-matched control mAb used for this labeling was the PE mouse IgG1, κ isotype control (BD, Cat# 555749, clone MOPC-21).<br><br>Human leukemia cells were identified using the following anti-human mAbs (all from BioLegend otherwise stated): anti-CD45 PE/Cy7 (Cat# 304016, clone HI30); anti-CD33 FITC (Cat# 303304, clone HIM3-4), PE or APC (Cat# 303404 and 303408, respectively; clone WM53); anti-CD34 (clone 581) FITC or APC (Cat# 343504 and 343510, respectively); anti-CD19 (clone HIB19) FITC, PE, PerCP/Cy5.5 or APC/Cy7 (Cat# 302206, 302208, 302230, and 302218, respectively); anti-CD10 APC (Cat# 312210, clone HI10a), CD1d PE (BD, Cat#

550255, clone 42.1).

T cells were labeled with the following mAbs from BioLegend: anti-mouse TCRβ chain (clone H57-597) PE, PerCP/Cy5.5 or APC/Cy7 (Cat# 109208, 109228, 109220, respectively) and anti-human TCRα/β-APC (Cat# 306718; clone IP26); CD3 (clone HIT3a) FITC, PerCP/Cy5.5, PE/Cy7, APC or APC/Cy7 (Cat# 300306, 300328, 300316, 300312, and 300318, respectively); CD4 (clone RPA-T4) FITC, PE/Cy7 or APC (Cat# 300506, 300512, and 300314, respectively); CD8 (clone SK1) PE, PE-Cy7, APC or APC/Cy7 (Cat# 344706, 344712, 344722, and 344714, respectively); CD69 APC (Cat# 310910, clone FN50).

Mouse cells were detected with anti-mouse CD45 APC/Cy7 (BioLegend, Cat#103116, clone 30-F11 or V500 (BD, Cat# 561487, clone 30-F11).
Circulating human B cells, monocytes and cDCs were identified using anti-human CD19 FITC, PE or PerCP/Cy5.5 (BioLegend, Cat# 302206, 302208 and 302230, respectively; clone HIB19), anti-human CD20-PE/Cy7 (BioLegend, Cat#302312, clone 2H7), anti-human CD14-PerCP/Cy5.5 (BioLegend, Cat# 301824, clone M5E2), and anti-CD11c-APC (BioLegend, Cat# 301824, clone 3.9).

The monoclonal antibodies used only in supplementary figures for Jurkat 76 β2m- and T cells phenotypical characterization were: HLA-ABC APC (Cat# 311410, clone W6/32); β2m PE (Cat# 316306, clone 2M2); CD62L PE/Cy7 (Cat# 304822, clone DREG-56); CD45RA FITC (Cat# 983002, clone HI100); CCR7 PE (Cat# 353204, clone G043H7); CD95 PE (Cat# 305608, clone DX2); CD4 V500 (BD, Cat# 560768, clone RPTA-4).

The purified anti-CD1c (Santa Cruz Biotechnology Inc, Cat# SC-53192, clone M241) and NA/LE mouse IgG1κ isotype control (BD, Cat# 553447, clone 107.3) mAbs were used in the in vitro TCR-transduced T cell assays.

The mouse anti-human IFN-γ mAb (Invitrogen, Cat# M700A, clone 2G1) and the mouse anti-human IFN-γ biotin-labeled mAb (Invitrogen, Cat# M701B, clone B133.5) were used in the ELISA assays.

Validation

No customized antibodies were used.
All antibodies used flow cytometry are from commercial sources and they were purchased from Santa Cruz Biotechnology Inc., BioLegend, BD. Validation data are available on the manufactures' web site and datasheets.

anti-human CD1c PE (Santa Cruz Biotechnology Inc., Cat# sc-18886, clone L161)
https://www.scbt.com/p/cd1c-antibody-l161

anti-human CD1c PE (BioLegend, Cat# 331506, clone L161)
https://www.biolegend.com/en-us/products/pe-anti-human-cd1c-antibody-4847?GroupID=BLG10089

PE mouse IgG1, κ isotype control (BD, Cat# 555749, clone MOPC-21).
https://www.bdbiosciences.com/en-us/products/reagents/flow-cytometry-reagents/research-reagents/flow-cytometry-controls-and-lysates/pe-mouse-igg1-isotype-control.555749

anti-human CD45 PE/Cy7 (BioLegend, Cat# 304016, clone HI30)
https://www.biolegend.com/nl-be/products/pe-cyanine7-anti-human-cd45-antibody-1915

anti-human CD33 FITC (BioLegend, Cat# 303304, clone HIM3-4)
https://www.biolegend.com/en-us/products/fitc-anti-human-cd33-antibody-726

anti-human CD33 PE or APC (Biolegend, Cat# 303404 and 303408, respectively; clone WM53)
https://www.biolegend.com/fr-ch/products/pe-anti-human-cd33-antibody-878
https://www.biolegend.com/en-us/products/apc-anti-human-cd33-antibody-877?GroupID=BLG10601

anti-human CD34 (clone 581) FITC or APC (BioLegend, Cat# 343504 and 343510, respectively)
https://www.biolegend.com/en-us/products/fitc-anti-human-cd34-antibody-6032?GroupID=BLG7551
https://www.biolegend.com/en-us/products/apc-anti-human-cd34-antibody-6090?GroupID=BLG7551

anti-human CD19 (clone HIB19) FITC, PE, PerCP/Cy5.5 or APC/Cy7 (BioLegend, Cat# 302206, 302208, 302230, and 302218, respectively)
https://www.biolegend.com/en-us/products/fitc-anti-human-cd19-antibody-717?GroupID=BLG10095
https://www.biolegend.com/en-us/search-results/pe-anti-human-cd19-antibody-719
https://www.biolegend.com/en-us/search-results/percp-cyanine5-5-anti-human-cd19-antibody-4226
https://www.biolegend.com/en-us/search-results/apc-cyanine7-anti-human-cd19-antibody-1910

anti- human CD10 APC (BioLegend, Cat# 312210, clone HI10a)
https://www.biolegend.com/en-us/products/apc-anti-human-cd10-antibody-2484?GroupID=BLG5905

anti-human CD1d PE (BD, Cat# 550255, clone 42.1).
https://www.bdbiosciences.com/en-us/products/reagents/flow-cytometry-reagents/research-reagents/single-color-antibodies-ruo/pe-mouse-anti-human-cd1d.550255
anti-mouse TCRβ chain (clone H57-597) PE, PerCP/Cy5.5 or APC/Cy7 (BioLegend, Cat# 109208, 109228, 109220, respectively)
https://www.biolegend.com/en-us/search-results/pe-anti-mouse-tcr-beta-chain-antibody-272
https://www.biolegend.com/en-us/search-results/percp-cyanine5-5-anti-mouse-tcr-beta-chain-antibody-5603

https://www.biolegend.com/en-us/search-results/apc-cyanine7-anti-mouse-tcr-beta-chain-antibody-4137

anti-human TCRα/β-APC (BioLegend, Cat# 306718; clone IP26)
https://www.biolegend.com/en-us/search-results/apc-anti-human-tcr-alpha-beta-antibody-6704

anti-human CD3 (clone HIT3a) FITC, PerCP/Cy5.5, PE/Cy7, APC or APC/Cy7 (BioLegend, Cat# 300306, 300328, 300316, 300312, and 300318, respectively)
https://www.biolegend.com/fr-fr/products/fitc-anti-human-cd3-antibody-751
https://www.biolegend.com/fr-fr/search-results/percp-cyanine5-5-anti-human-cd3-antibody-5613
https://www.biolegend.com/fr-fr/search-results/pe-cyanine7-anti-human-cd3-antibody-1913
https://www.biolegend.com/fr-fr/search-results/apc-anti-human-cd3-antibody-749
https://www.biolegend.com/fr-fr/search-results/apc-cyanine7-anti-human-cd3-antibody-1912

anti-human CD4 (clone RPA-T4) FITC, PE/Cy7 or APC (BioLegend, Cat# 300506, 300512, and 300314, respectively)
https://www.biolegend.com/en-us/products/fitc-anti-human-cd4-antibody-825?GroupID=BLG5901
https://www.biolegend.com/en-us/search-results/pe-cyanine7-anti-human-cd4-antibody-829
https://www.biolegend.com/en-us/search-results/apc-anti-human-cd4-antibody-823

anti-human CD4-V500 (BD, Cat# 560768, clone RPTA-4)
https://www.bdbiosciences.com/en-us/products/reagents/flow-cytometry-reagents/research-reagents/single-color-antibodies-ruo/v500-mouse-anti-human-cd4.560768

anti-human CD8 (clone SK1) PE, PE-Cy7, APC or APC/Cy7 (BioLegend, Cat# 344706, 344712, 344722, and 344714, respectively)
https://www.biolegend.com/en-us/products/pe-anti-human-cd8-antibody-6247?GroupID=BLG10167
https://www.biolegend.com/en-us/search-results/pe-cyanine7-anti-human-cd8-antibody-6390
https://www.biolegend.com/en-us/search-results/apc-anti-human-cd8-antibody-6531
https://www.biolegend.com/en-us/search-results/apc-cyanine7-anti-human-cd8-antibody-6391

anti-human CD69 APC (BioLegend, Cat# 310910, clone FN50)
https://www.biolegend.com/en-us/products/apc-anti-human-cd69-antibody-1674?GroupID=BLG10036

anti-human HLA-ABC APC (BioLegend, Cat# 311410, clone W6/32)
https://www.biolegend.com/en-us/products/apc-anti-human-hla-a-b-c-antibody-1870

anti-human β2m PE (BioLegend, Cat# 316306, clone 2M2)
https://www.biolegend.com/en-us/products/pe-anti-human-beta2-microglobulin-antibody-3080?GroupID=BLG4187

anti-human CD62L PE/Cy7 (BioLegend, Cat# 304822, clone DREG-56)
https://www.biolegend.com/nl-be/products/pe-cyanine7-anti-human-cd62l-antibody-3944?GroupID=BLG10270

anti-human CD45RA FITC (BioLegend, Cat# 983002, clone HI100)
https://www.biolegend.com/en-us/products/fitc-anti-human-cd45ra-antibody-13994

anti-human CCR7 PE (BioLegend, Cat# 353204, clone G043H7)
https://www.biolegend.com/en-us/products/pe-anti-human-cd197-ccr7-antibody-7498?GroupID=BLG9611

anti-human CD95 PE (BioLegend, Cat# 305608, clone DX2)
https://www.biolegend.com/en-us/products/pe-anti-human-cd95-fas-antibody-643

anti-mouse CD45 APC/Cy7 (BioLegend, Cat#103116, clone 30-F11)
https://www.biolegend.com/en-us/products/apc-cyanine7-anti-mouse-cd45-antibody-2530?GroupID=BLG1932

anti-mouse V500 (BD, Cat# 561487, clone 30-F11)
https://www.bdbiosciences.com/en-us/products/reagents/flow-cytometry-reagents/research-reagents/single-color-antibodies-ruo/v500-rat-anti-mouse-cd45.561487

anti-human CD20-PE/Cy7 (BioLegend, Cat#302312, clone 2H7)
https://www.biolegend.com/en-us/products/pe-cyanine7-anti-human-cd20-antibody-1902?GroupID=BLG7904

anti-human CD14-PerCP/Cy5.5 (BioLegend, Cat# 301824, clone M5E2)
https://www.biolegend.com/en-us/search-results/percp-cyanine5-5-anti-human-cd14-antibody-4223

anti-CD11c-APC (BioLegend, Cat# 301824, clone 3.9)
https://www.biolegend.com/en-us/search-results/apc-anti-human-cd11c-antibody-2865?GroupID=GROUP28

Purified antibodies used in the bioassays were purchased from Santa Cruz Biotechnology Inc, or BD and were validated by the manufacturers:
purified anti-CD1c (Santa Cruz Biotechnology Inc, Cat# SC-53192, clone M241)

https://www.scbt.com/it/p/cd1c-antibody-m241

purified NA/LE mouse IgG1κ isotype control (BD, Cat# 553447, clone 107.3)
https://www.bdbiosciences.com/en-us/products/reagents/immunoassay-reagents/purified-na-le-mouse-igg1-isotype-control.554721

The antibodies used for ELISA assays were purchased from Invitrogen and they were validated by the manufacturer:

mouse anti-human IFN-γ mAb (Invitrogen, Cat# M700A, clone 2G1)
https://www.thermofisher.com/antibody/product/IFN-gamma-Antibody-clone-2G1-Monoclonal/M700A

mouse anti-human IFN-γ biotin-labeled mAb (Invitrogen, Cat# M701B, clone B133.5)
https://www.thermofisher.com/antibody/product/IFN-gamma-Antibody-clone-B133-5-Monoclonal/M701B

Eukaryotic cell lines

Policy information about cell lines

Cell line source(s)	Jurkat 76 cells were provided by Prof. H.J. Stauss, University College London; the original commercial source was not retrievable.<br>MOLT-4, K562-WT, THP-1-WT and 293T cells were purchased by American Type Culture Collection, Manassas, VA (ATCC);<br>K562 transfected to express CD1c were provided by Prof. D.B. Moody, Brigham and Women's Hospital, Harvard Medical School, Boston. The original commercial source was not retrievable (ref de Jong et al, Nat. Immunol 2010);<br>THP-1- transfected to express CD1c and CCRF-SB were provided by Prof. Gennaro De Libero, University of Basel and University Hospital, Basel, Switzerland. The original commercial source was the ATCC.<br>NALM-6 and THP-1 expressing the Gaussia LUCIA were provided by Dr. Monica Casucci, IRCCS San Raffaele Scientific Institute, Milan, Italy; The original commercial source was the ATCC.<br>C1R-CD1c were provided by Prof. S. Porcelli, Albert Einstein College of Medicine, New York; The original commercial source was not retrievable.<br>MOLM-13 were kindly provided by Dr. R. Bernardi, IRCCS San Raffaele Scientific Institute and then transduced with a lentiviral vector expressing the Gaussia LUCIA. The original commercial source was not retrievable.<br>Luciferase Lucia-expressing THP-1, NALM-6 and MOLM-13 cells were transduced with a LV encoding CD1c.
Authentication	None of the utilized cell lines were authenticated.
Mycoplasma contamination	All cell lines tested negative for mycoplasma
Commonly misidentified lines (See ICLAC register)	None

Animals and other organisms

Policy information about studies involving animals; ARRIVE guidelines recommended for reporting animal research

Laboratory animals	8-week-old male NOD.Cg-Prkdcscid IL- 2rgtm1Wjl/SzJ (NSG) mice.
Wild animals	The study did not involve wild animals
Field-collected samples	The study did not involve samples collected from the field
Ethics oversight	The study was approved by the San Raffaele Scientific Institute IACUC and by the Italian Ministry of Health (Rome, Italy) (protocols 678 and 1072).

Note that full information on the approval of the study protocol must also be provided in the manuscript.

Human research participants

Policy information about studies involving human research participants

Population characteristics	Primary leukemia samples were obtained from peripheral blood of adult and pediatric patients (m/f) at diagnosis. The age range of adult patients was between 25-88 years, while that of pediatric patients was 3-10 years.<br>Healthy volunteers were anonymous, but for them there were no covariate-relevant population characteristics to take in account for our study.
Recruitment	No potential self-selection bias is known. Patients were recruited at diagnosis, with a percentage of circulating blasts between 60%-97% of leukocytes to minimize manipulation following density gradient centrifugation. Moreover, only patients with CD1c expression on blasts with RFI ≥ 2.5 were recruited.<br>Healthy volunteers were recruited without any selection because the buffy coats distributed by the Transfusion Center of the San Raffaele Hospital are anonymous. Nevertheless, there were no covariate-relevant population characteristics to take in account.
Ethics oversight	The study protocol “CD1TARGET” was approved by the San Raffaele Ethics Committee on November 6th, 2018. The study

was conducted in accordance with the Declaration of Helsinki principles. All participants and/or their legal guardians provided written informed consent. Healthy donors were recruited upon inform consent signing.

Note that full information on the approval of the study protocol must also be provided in the manuscript.

Flow Cytometry

Plots

Confirm that:

- [x] The axis labels state the marker and fluorochrome used (e.g. CD4-FITC).
- [x] The axis scales are clearly visible. Include numbers along axes only for bottom left plot of group (a 'group' is an analysis of identical markers).
- [x] All plots are contour plots with outliers or pseudocolor plots.
- [ ] A numerical value for number of cells or percentage (with statistics) is provided.

Methodology

Sample preparation

PBMCs from buffy coat of healthy volunteers or from peripheral blood of leukemia patients were obtained by density gradient centrifugation. CD4+ T cells were isolated using CD4 Isolation Kit (Miltenyi Biotec). T cells were activated with Dynabeads® human T-Activator CD3/CD28 (Thermofisher) at a 3:1 bead:cell ratio for 2 days, then transduced with the LV and further expanded until day 18-20 before use (or cryopreservation). Normal CD1c+ APCs were purified from PBMCs cells of healthy volunteers using anti-CD19 (B cells), anti-CD14 (monocytes) or anti-CD11c-APC mAb and anti-APC (cDCs) immunomagnetic beads according to the manufacturer's protocols (Miltenyi Biotec). Mo-DCs were differentiated in vitro from purified CD14+ monocytes in the presence of GM-CSF and IL-4 (Peprotech) as previously described.
Prior to labeling: mouse peripheral blood samples were treated with ACK lysis buffer to lyse RBCs the cells; Bone marrow cells simply flushed out from femurs of mice; mouse hepatic cells were isolated by using Percoll solution and subjected to red blood cells lyses with ACK

Before surface staining, to block unspecific mAbs binding, human cells and mouse cells were incubated 20 minutes at RT either in PBS 1X + 10 % NHS or in 150 ng/ml mouse IgG, respectively. Then the cells were stained with the indicated mAbs diluted in FACS-wash (PBS-2% FBS 0.02%NaN3) for 15 minutes on ice in the dark. Cells were next washed 3 times with FACS-wash (1500 rpm, 4°C, 5 minutes) and 100 ng/ml DAPI was added to exclude dead cells.

Instrument

FACS Canto II (BD) flow cytometer

Software

Flow cytometry samples were collected using BD FACSDiva (v8.0.2) and analyzed using FlowJo (v10.6)

Cell population abundance

Typically labelled 0.5-2x10^6 cells.

Gating strategy

- Jurkat 76 b2m cells were subsequently gated in by physical parameters FSC-A vs SSC-A, singlets (FSC-A vs FSC-H) and viability using DAPI vs FSC-A. CD69 upregulation was further gated in mouse-TCRb-expressing GFP+ transduced cells
- To characterize transduced primary T cells, lymphocytes were gated in by physical parameters FSC-A vs SSC-A, singlets (FSC-A vs FSC-H) and viability using DAPI vs FSC-A The transduced T cells were identified by gating CD3 vs FSC-A, followed by mouse-TCRb histograms. Finally, T cells were gated in mouse-TCRb vs FSC-A to analyze the endogenous human-TCR expression by histogram.
- For in vitro killing assays with T cells, effector and target cells were subsequently gated by physical parameters FSC-A vs SSC-A and viability using DAPI vs FSC-A. T cells were identified as CD3+ whereas targets cells were defined (depending on the cell type) as follow: CD33+ for THP1, MOLM, K562, AML-11, AML-33; CD34+ for AML-32, AML-42, AML-48; CD19+ for CCRF, C1R, B cells; CD10+ for NALM and B-ALL-31; CD14 for monocytes; CD11c for monocytes and DCs.
-For in vivo experiments, human T cells were subsequently gated by physical parameters FSC-A vs SSC-A, singlets (FSC-A vs FSC-H), viability using DAPI vs FSC-A, human CD45+ mouse-CD45- cells, mouse-TCRb+ (TCR-transduced T cells) human-CD3+ (all human T cells). Leukemia cells were defiened as follow: GFP+ for THP-1, human CD19+ for NALM-6 and human CD33 for NALM-6.

- [x] Tick this box to confirm that a figure exemplifying the gating strategy is provided in the Supplementary Information.
